# Supplementary material for: Interfaces between hexagonal and cubic oxides and their structure alternatives
Source: Nat Commun. 2017 Nov 14;8:1474. doi: 10.1038/s41467-017-01655-5 (PMC5684368; doi:10.1038/s41467-017-01655-5)
Supplement: Supplementary file 1 — Supplementary Information [file 41467_2017_1655_MOESM1_ESM.pdf]

Supplementary Note1. Generation of the diagram in Fig. 1.

The generation of the diagram in Fig. 1 is based on a series of XRD results and the corresponding *in-situ* RHEED patterns from the ZnO films as shown in Supplementary Figure 1. Supplementary Figure 1a shows the evolution of the growth orientation through tailoring the growth temperature while fixing the O_2 pressure at 1×10^{-5} mbar. It is clearly seen that the growth orientation of the film is along (001) ZnO when the growth temperature is at about 450K, as marked by the red arrow in Supplementary Figure 1a. When the growth temperature is up to or larger than 600K, the growth orientation becomes along (100) ZnO, as marked by the blue arrow in Supplementary Figure 1a. Similarly, the growth orientations can be modified by the O_2 pressure with fixing the growth temperature, as demonstrated in Supplementary Figure 1(b-d). Supplementary Figure 1(e-h) display the *in-situ* RHEED patterns, showing the growth orientation transformation, corresponding to the XRD results in Supplementary Figure 1(a-d). A diagram of growth orientation as a function of growth temperature and oxygen pressure can thus be generated, as shown in Fig. 1 in the main text.

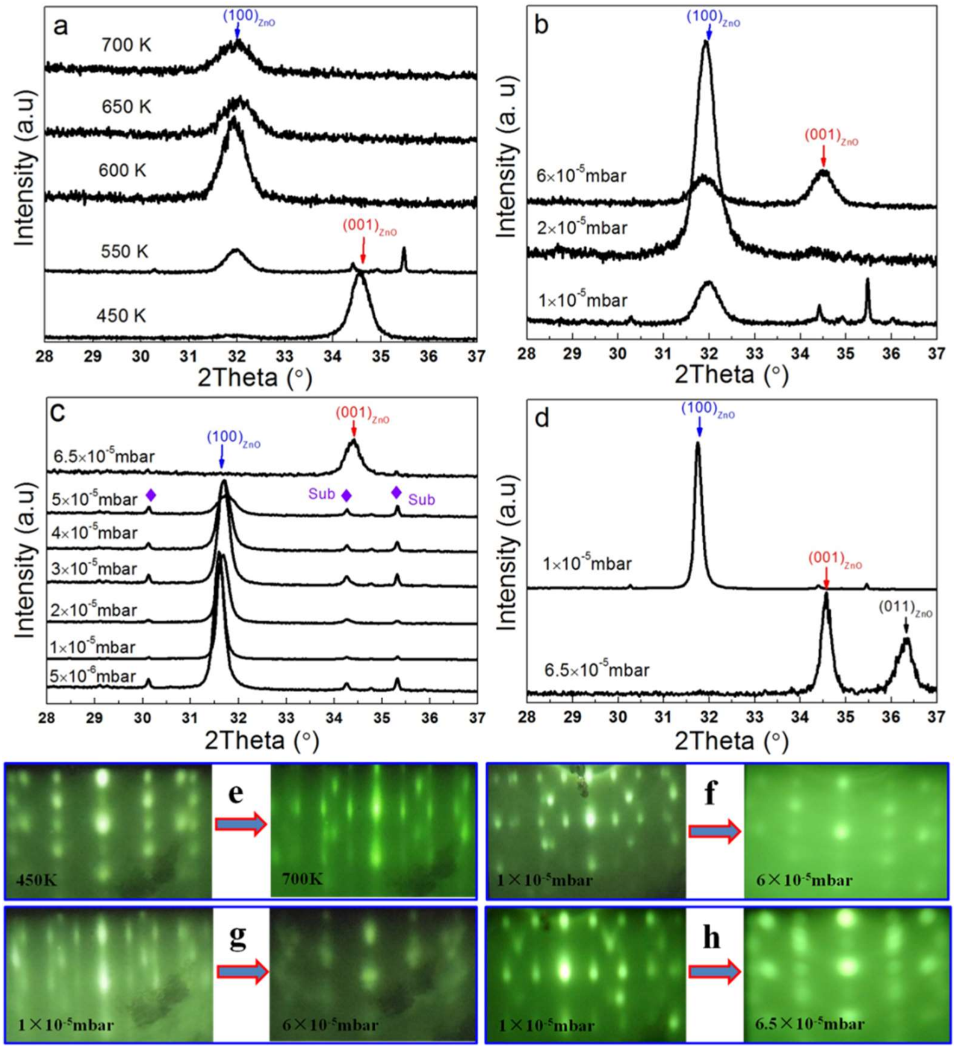

Supplementary Figure 1. X-ray Diffraction (XRD) results and in-situ reflection high energy electron diffraction (RHEED) patterns from the ZnO films under different growth conditions. (a) Varying growth temperature from 450K to 700K while fixing the O_2 pressure at 1×10^{-5} mbar; (b), (c) and (d) Varying the O_2 pressure while fixing the growth temperature at about 540K, 600K and 630K, respectively; (e-h) In-situ RHEED patterns, showing the growth orientation transformation, corresponding to the XRD results (a-d).

Supplementary Note 2. 3D view of the interface.

Supplementary Figure 2(a-d) show the three-dimensional (3D) view of atomic models for the interface structures of c-ZnO domain I, c-ZnO domain II, m-ZnO domain I and m-ZnO domain II, respectively.

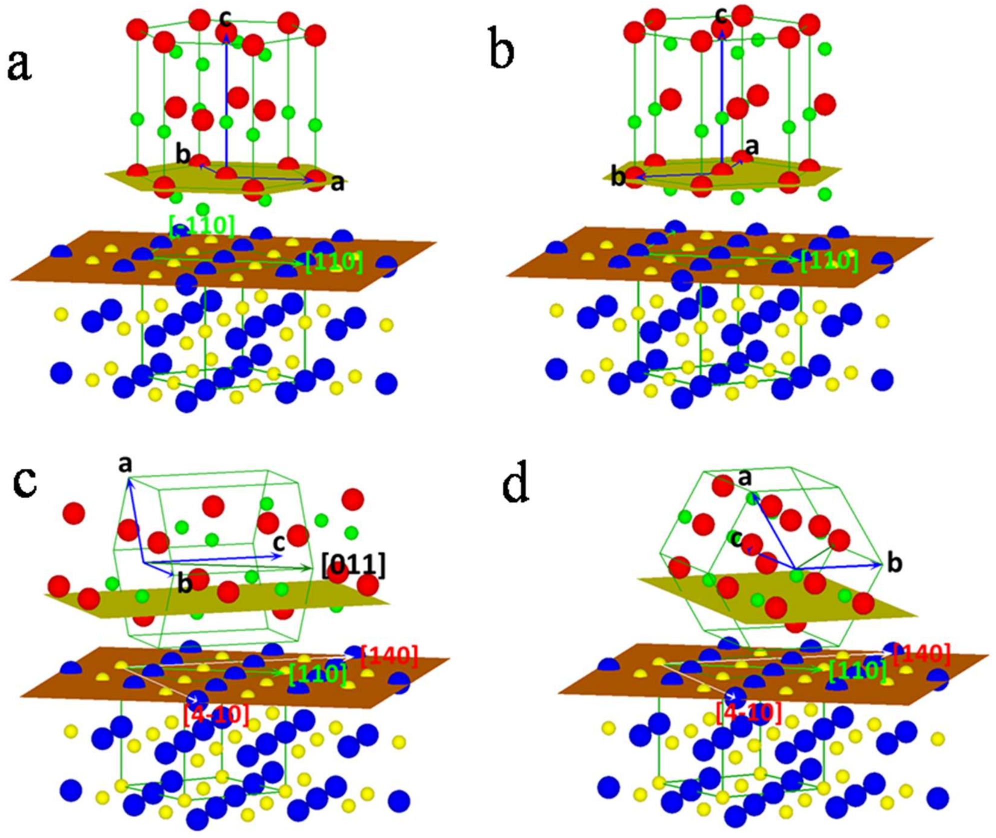

Supplementary Figure 2. Three-dimensional (3D) view for the interface structure. (a) c-ZnO domain I. (b) c-ZnO domain II. (c) m-ZnO domain I. (d) m-ZnO domain II. Red, green, blue and yellow spheres represent Zn, O in ZnO, Mg and O in MgO, respectively. Green lines outline the lattice of ZnO and MgO. Orange plane shows MgO (001) plane. Green-yellow plane shows ZnO (001) plane in (a,b), and ZnO (100) plane in (c,d), respectively.

Supplementary Note 3. The orientation relationship for m-ZnO film by STEM simulations.

Because the interface structures of m-ZnO films are more complicated than those of c-ZnO films, it is necessary to further analyze the STEM images from the m-ZnO films. Supplementary Figures 3 (a-d) show the calculated STEM-HAADF images for ZnO with the beam along [011] (a), [0-11] (b), [08-3] (c), [083] (d) directions, respectively. The features of these STEM-HAADF patterns are consistent with the experimental observations (Figs. 4a and 4b in the main text). Supplementary Figure 3e and 3f are corresponding atomic models from the side and top views, respectively.

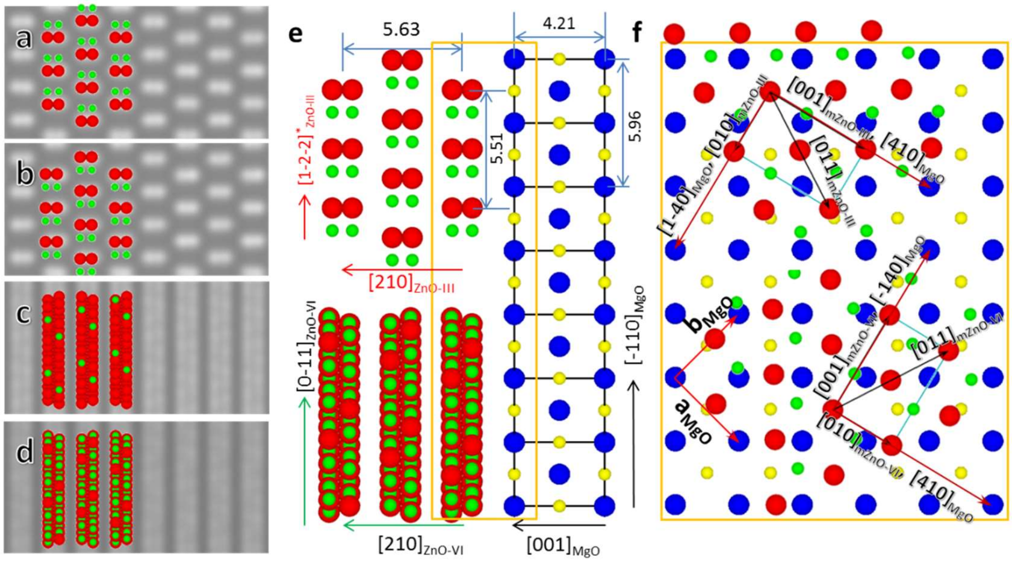

Supplementary Figure 3. (a-d) Calculated scanning transmission electron microscopy (STEM) -_high-angle annular dark-field (HAADF) images for ZnO with the beam along (a) [011], (b) [0-11], (c) [08-3], and (d) [083] directions, respectively. The convergent angle is 21 mrad, and the collection angle is 67-275 mrad. The corresponding atomic projections are embedded in the images. Projection of ZnO along [0-11] (upper panel, domain III) and [083] (lower panel, domain VI) along with MgO [110] projection. (f) Top view with one layer of ZnO (100) on one layer of MgO (001) substrate for ZnO domain III (top) and domain VI (bottom). The model in (f) is obtained by rotating 90° clockwise from the interface area outlined by the orange rectangle in (e). The ZnO-VI domain (see the arrows) is rotated 90° from that of ZnO-III domain.

Supplementary Note 4. The interface relationship for m-ZnO by RHEED.

The relationship between the SAED patterns and the angle of the rotation along the normal of the substrate can be further probed in the RHEED patterns, as shown in Supplementary Figure 4a and 4b. The RHEED pattern from the 120-min growth homoepitaxial ZnO film (Supplementary Figure 4a), shows the regular hexagon matrix. The image was captured along the $[001]_{ZnO}$ direction. After rotating ±(31±2°), and arriving at the [011] or $[0\text{-}11]_{ZnO}$ direction, there would appear a compressed hexagonal matrix in the RHEED patterns, as shown in Supplementary Figure 4b. This is very similar to the SAED pattern discussed in the main text. The lower images, Supplementary Figure 4c and 4d, are the corresponding atomic models. Note that the rotational angle between the two azimuths is about 62° and that the RHEED patterns along [011] ZnO and [0-11] ZnO directions are identical.

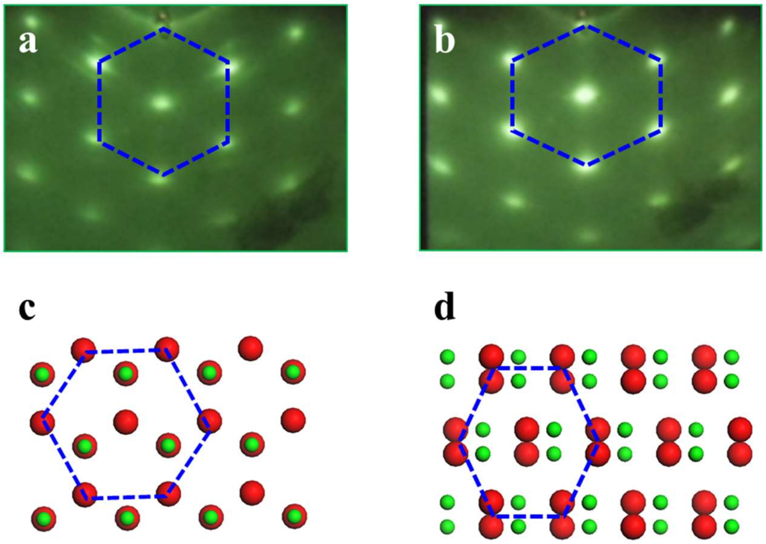

Supplementary Figure 4. (a) and (b) In-situ reflection high energy electron diffraction (RHEED) patterns from the homoepitaxy of m-plane ZnO film along [001] azimuth and [011] azimuth directions, respectively; (c) and (d) the corresponding atomic models from the side view along the [001] azimuth and [011] azimuth directions, respectively.

Supplementary Note 5. Occurrence of rotational domains for c-ZnO and m-ZnO from theoretical points of view

Experimentally, we found that there are two rotational domains in the c(001)-ZnO films grown on the (001) MgO substrate; while four domains appear in the m(100)-ZnO films grown on the same substrate surface. This is in agreement with the theoretical prediction based on the mismatch of rotational symmetry at the interface using the formula: [1]

$$N_{RD} = \frac{lcm\ (n, m)}{m},$$ (Supplementary Equation 1)

where N_{RD} is the number of rotational domains expected in the grown epilayer, n denotes the Cn rotational symmetry of the substrate crystal with rotation angles $\Phi_i=2\pi/n$, m denotes the C_m rotational symmetry of the epilayer crystal with rotation angles $\Phi_j=2\pi/m$, and $lcm(n, m) = k/(i/n+j/m)$ is the least common multiple of n and m, where i, j and k are integers.

In the c-ZnO / MgO case, the cubic MgO and hexagonal ZnO have rotational symmetries of C_4 $(n=4)$ and C_6 $(m=6)$, respectively. And $lcm\ (4,6)=12$. Therefore, $N_{RD}=12/6=2$, *i.e.* two rotational domains are expected in the c-ZnO epilayer grown on the MgO substrate, which is exactly what our results have shown.

In the m-ZnO / MgO case, the [010] (or [0-10]) direction is parallel to one of the MgO <410> directions. There are eight equivalent in-plane <410> directions on the MgO (001) surface, leading to a rotational symmetry of C_8 $(n=8)$ while the m-ZnO film is of a rotational symmetry of C_2 $(m=2)$. As $lcm\ (8,2)=8$, we obtain $N_{RD}=8/2=4$, *i.e.* four rotational domains are expected in the m-ZnO epilayer grown on the MgO substrate, which is also what our results have shown.

Supplementary Note 6. Termination of the c-ZnO film.

For the c-ZnO film, the termination at the interface could be either oxygen-plane or zinc-plane. To determine the termination, we simultaneously acquired STEM high angle annular dark field (HAADF) and annular bright field (ABF) images for c-ZnO, as shown in Supplementary Figure 5a and 5b, respectively. The insets show the magnified images from the area outlined by the rectangles with the atomic projection of $[100]_{cZnO\text{-}I}$. While O contrast is weak and blur near the interface due to the strain, they are clearly seen on the right side of the Zn atoms in the magnified ABF image, indicating the ZnO film is along the [001] direction, *e.g.* O is closer to the interface than Zn at the interface, which is consistent with our first principle calculations that the O-termination at the interface yields a lower interface energy (see discussions later). Supplementary Figure 5e and 5f show simultaneously acquired STEM –HAADF and STEM-ABF images for the c-ZnO film away from the interface. The sharper O contrast in the ABF image can be seen as the film in this area is free from defects.

Supplementary Figure 5c shows an inverse FFT images by applying an aperture in $020_{ZnO}/\text{-}220_{MgO}$ spots of the FFT (inset) of STEM-HAADF image in Supplementary Figure 5a. Horizontal fringes with a spacing of about 1.41 Å in the ZnO film and 1.49 Å in the MgO substrate are present. Dislocations with an average spacing of about 2.3 nm are clearly seen, as marked by T. Note that $010_{ZnO}/\text{-}110_{MgO}$ spots are not suitable to show dislocations because the diffraction from $\text{-}110_{MgO}$ is extinct. Supplementary Figure 5d shows the schematic of the possible interface dislocation network (the top view, similar to Fig. 1d). If the ZnO layer at z=0 contacts with the MgO surface to form the interface (denoted as I1), it forms a centered rectangle coincident site lattice (CSL) with $17[120]_{ZnO}$ / $16[\text{-}110]_{MgO}$ (or 34 Zn / 32 Mg rows) in height and $11[100]_{ZnO}$ / $6[110]_{MgO}$ (11 Zn / 12 Mg columns) in width, where Zn (red) and Mg (blue) atoms are coincident, as marked by blue circles and blue lines. There are 17 Zn rows, while 16 Mg rows between two blue lines, indicating one extra Zn row per 16 Mg rows, resulting in the formation of the interface dislocations with Burgers vector b=[010] and dislocation line direction along [100], as shown by horizontal blue lines. In the interface, additional atoms are observed, as indicated by orange arrows in (a). This indicates that the MgO surface is not atomically flat, *e.g.* some surface may be terminated at MgO at z=0, while the others at z= ½. We thus observe mixed Mg and Zn atoms in the MgO [110] projection. The different MgO terminated surface would cause ZnO at z= ½ (green spheres) to contact with MgO to form the interface (denoted as I2). Similar dislocations would form at I2 but shift $3[\text{-}110]_{MgO}$ in terms of I1 interface dislocations, as shown by red lines in (d). Although we should only observe 1 dislocation per 8[-110]MgO, or 1 dislocation per 32 MgO (-220) lattice fringes when only one type interface is present, we would observe 2 dislocations per 8[-110]MgO, or 2 dislocation per 32 MgO (-220) lattice fringes when two types of interface present, *e.g.* along the beam direction. Therefore, we may observe 1 dislocation per 4[-110]MgO, or 1 dislocation per 16 MgO (-220) / 17 ZnO (020) lattice fringes (note, MgO [-110] length = $4d_{\text{-}220}$) when viewed along $[110]_{MgO}$ direction, consistent with the observation in (c). Similarly, there should be one interface dislocation per $6[110]_{MgO}$ with Burgers vector b=[100] because there are 12 Mg atoms while only 11 Zn atoms between two coincident sites horizontally. The dislocation line may not be straight because the CSL lattice lines (green lines) are not parallel to $[010]_{ZnO}$ direction.

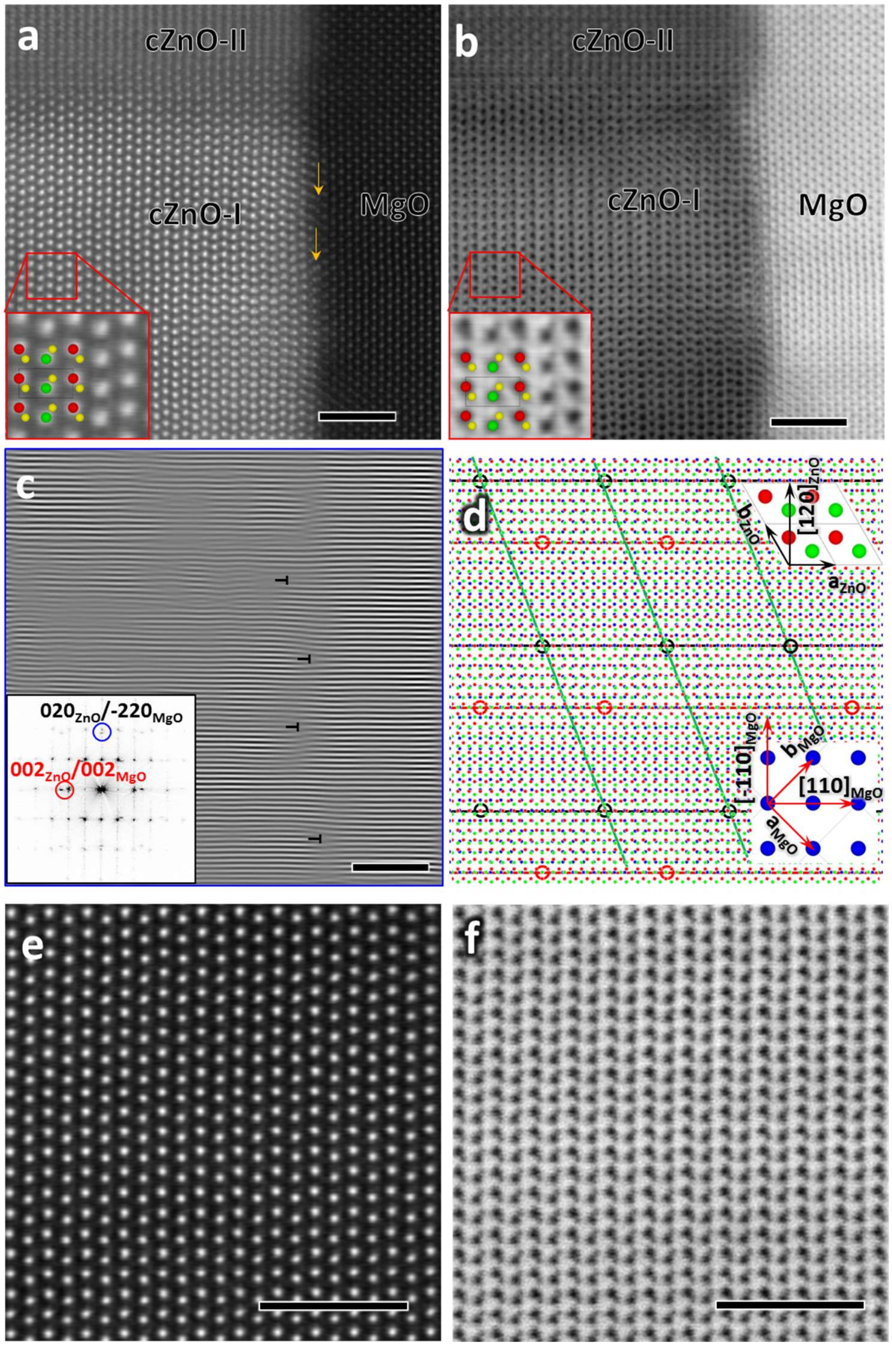

Supplementary Figure 5. Simultaneously acquired scanning transmission electron microscopy (STEM) high angle annular dark field (HAADF) (a) and annular bright field (ABF) (b) images for c-ZnO film on (001) MgO substrate. Scale bar, 2nm. The insets show the magnified images from the area outlined by the rectangles with the atomic projection of $[100]_{cZnO\text{-}I}$. Red, green and yellow spheres represent Zn at z=0, z= ½ and O atoms, respectively. (c) Inverse FFT images by applying an aperture in $020_{ZnO}/-220_{MgO}$ spots of the FFT (inset) of STEM-HAADF image in (a). Scale bar, 2nm. (d) Schematic of the possible interface dislocation network (top view similar to Fig. 2d in the main text). Blue, red and green spheres represent (001) Mg, (001) Zn at z=0 and z=1/2, respectively. Enlarged atoms with labels are shown on the right. O atoms are not drawn for clarity. (e,f) Simultaneously acquired STEM-HAADF (e) and STEM-ABF (f) images for c-ZnO from the film away from the interface with the MgO substrate on the right side. Scale bar, 2 nm. The crystal in this area is free from defects, thus has a sharper O contrast.

Supplementary Note 7. Domain boundary structures of the ZnO film.

Domain boundary structures are formed in regions of competing growth orientations. For c-ZnO, the domain boundary is sharp and nearly vertical to the interface, as indicated by the red dashlines in Supplementary Figure 6a. For m-ZnO (Supplementary Figure 6b), the domain boundary is not so obvious. The region between two red dash lines looks like the mixture of domain I and II, indicating the boundary is not parallel to the beam direction. Thus the domain I and II overlap in this region. The interphase in the region marked by two yellow dash lines has an atomic arrangement similar to that of MgO for the interface between m-ZnO domain I and MgO (upper part of Supplementary Figure 6b), as well as the interface between m-ZnO domain II and MgO (lower part of Supplementary Figures 6b and 6c). This indicates that the interphase has similar structure to cubic MgO, rather than hexagonal ZnO.

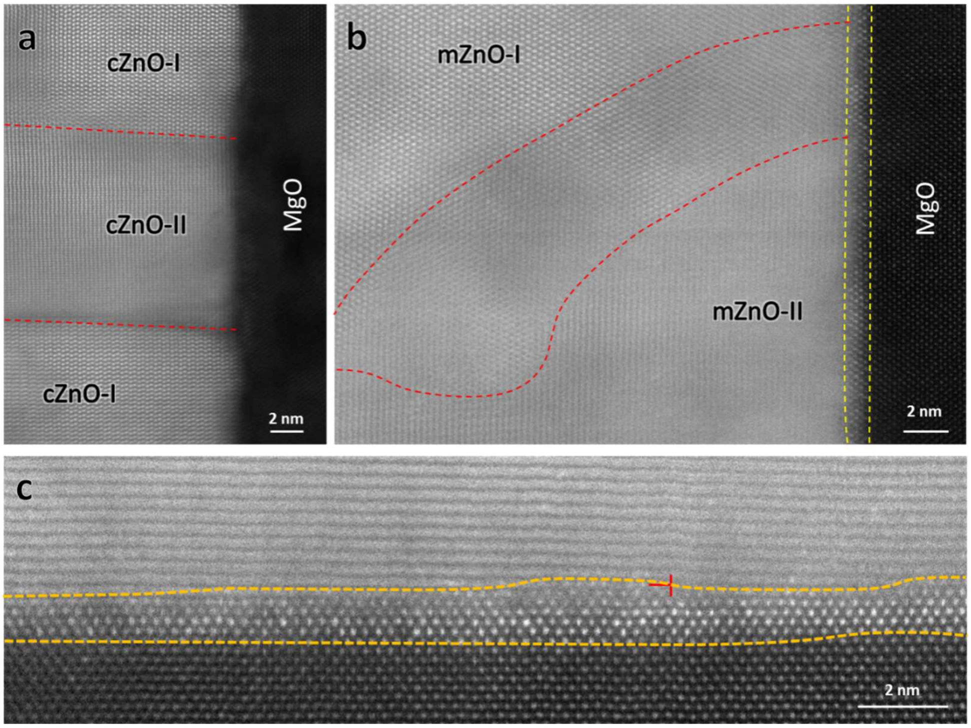

Supplementary Figure 6 (a) STEM-HAADF image for c-ZnO film, showing two domains. (b, c) STEM-HAADF images for m-ZnO film. The red dash lines outline the boundary between domains. The yellow dash lines mark the inter phase between the m-ZnO film and the MgO substrate.

Supplementary Note 8. Point defects at the interface of c-ZnO clarified by the STEM-HAADF image.

The image contrast acquired by HAADF-STEM is approximately proportional to $Z^{1.7}$ (Z: atomic number). Because the atom of Mg (Z=12) is much lighter than Zn (Z=30), the contrast of Mg is rather dark, which makes it difficult to be observed. We have enhanced the contrast of Fig. 2a with false color and plot the peak intensities in 3D and profile (Supplementary Figure 7) to confirm the point defects at the interface.

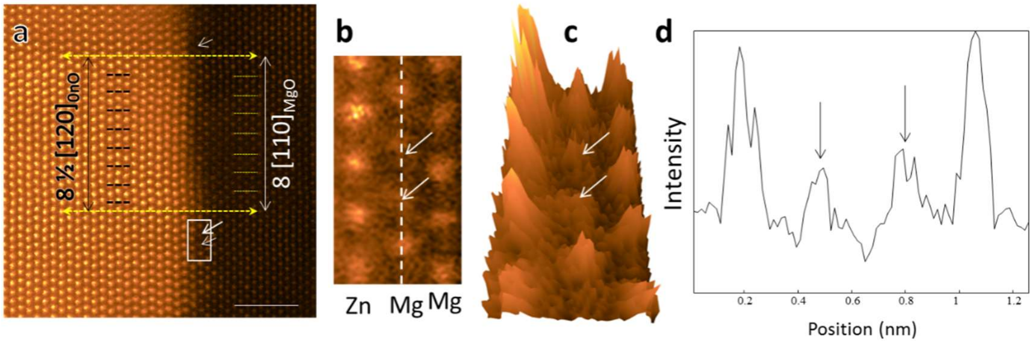

Supplementary Figure 7. (a) scanning transmission electron microscopy (STEM)-_high-angle annular dark-field (HAADF) image with false color. (b) Magnified image from the rectangle in (a). (c) 3D perspective view of (b). The peaks indicated by the arrows are apparently weaker than their neighbors. (d) Intensity profile from the vertical scan line shown in (b).

Supplementary Note 9. Lattice parameters estimated from RHEED patterns.

Supplementary Figure 8a and 8b show the in-situ RHEED patterns from the c-ZnO and m-ZnO after a 10 min growth. Supplementary Figure 8c and 8d are the corresponding atomic models. It is clear that every pattern shows two sets of reciprocal space diffraction images (as labeled by the rectangle or hexagon in Supplementary Figure 8a and 8b). The distances between the spots reflect the lattice parameters in reciprocal space. Therefore, through calculating the ratios of the reciprocal space lattice value, we can estimate the changes in the real lattice parameters of the film. In Supplementary Figure 8a, the ratios of $(\frac{c_{ZnO}}{2})^*/(\frac{a_{ZnO}}{2})^*$ and $c^*_{ZnO}/(\frac{\sqrt{3}}{2}a_{ZnO})^*$ are 1/1.63 and 1/1.87, respectively, while in Fig. S5b, the ration value of $c^*_{ZnO}/(\frac{\sqrt{3}}{2}a_{ZnO})^*$ is 1/1.86. These values are very close to the theoretical results ($(\frac{c_{ZnO}}{2})^*/(\frac{a_{ZnO}}{2})^*$ =a/c=1/1.60, $c^*_{ZnO}/(\frac{\sqrt{3}}{2}a_{ZnO})^* = (\frac{\sqrt{3}}{2}a_{ZnO})/c_{ZnO}$ =1/1.85). These results indicate that the lattice parameters in the film are nearly equal to those in the bulk, in good agreement with the results observed by STEM.

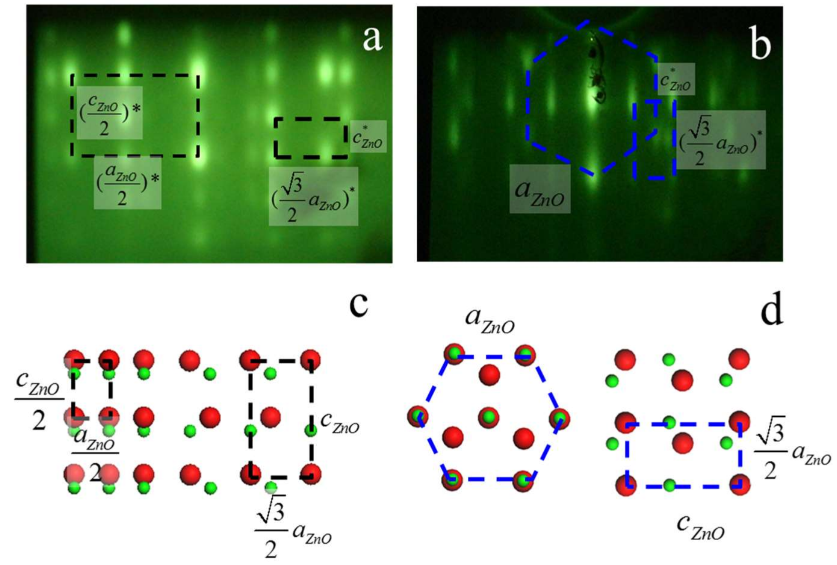

Supplementary Figure 8. Reflection high energy electron diffraction (RHEED) patterns from (a) c-ZnO and (b) m-ZnO; (c) is an atomic model of the pattern in (a) while (d) corresponds to the pattern in (b).

Supplementary Note 10. EELS analysis for the transition from ZnO to MgO.

The c- and m-ZnO films show distinguished interfaces with the substrate of MgO. STEM images (Fig. 2 and Fig. 4) suggest that the interface for c-ZnO is sharp, while for m-ZnO it is rough. This morphological difference could originate from the buffer layer of the m-ZnO. A transitional interphase region with cubic structure at the interface is due to the Zn atoms diffusion, as illustrated by the orange dashed lines in Figs. 4a and 4b. The gradual decrease of the intensity from the ZnO film to the MgO substrate (over 5-6 atomic layers) indicates that there exists an interphase $Zn_xMg_{1-x}O$ structure (x decreases from the ZnO side to the MgO side). This result can be further confirmed by the EELS spectrum image, as illustrated in Supplementary Figure 9. Supplementary Figure 9a is the STEM-HAADF survey image. The green line across the interface marks the scan line (position from 0 nm to 8.1 nm). Supplementary Figure 9b and 9c show the EELS spectra at different positions from the spectrum image for O K-edge, and Zn L_{32} and Mg-K edges, respectively. Only Zn L_{32} edges are present until the probe position at p=3.5 nm (hereafter p denotes position). The Mg K edge appears when the probe reaches p=4.1 nm. There are both Zn L_{32} and Mg K edges in the interface area between 4.1 nm and 5.1 nm, as shown in Supplementary Figure 9c. The Zn L_{32} disappears after the probe moves beyond 5.1 nm. Similar to these observations, in the O K-edge EELS spectrum, the peak at the 550 eV position derived from the MgO signal (marked by red vertical dash line in Supplementary Figure 9b) becomes visible at p=4.1 nm, and increases as the probe moves right. Supplementary Figure 9d shows the relative composition of Zn and Mg calculated from the EELS spectrum image based on multiple linear least-squares fittings with the reference spectra at the p=0 nm and p=8 nm. The composition of Mg starts to increase at about p=3.7 nm, and reaches 100% at about p=5.3 nm, indicating that the thickness of the $Zn_xMg_{1-x}O$ interphase is 1.6 nm or less. Moreover, when the ZnO film is aligned along [011] (Fig. 4a) and [0-83] (Fig. 4b), the interphase shows the same atomic projection as MgO, indicating that it has a structure similar to cubic MgO, rather than hexagonal ZnO. Remarkably, we find that Zn atoms permeating the 5-6 atomic layers of the initial surface of the substrate, are responsible for forming the interphase, rather than Mg atoms permeating ZnO thin films. This is due to the easy desorption of Mg atoms from the MgO surface[1]. Due to the differences of growth directions, the interface structures are distinct. The interface structure of c-ZnO is smooth with no buffer layer, while that of the m-ZnO is rough, with a buffer layer.

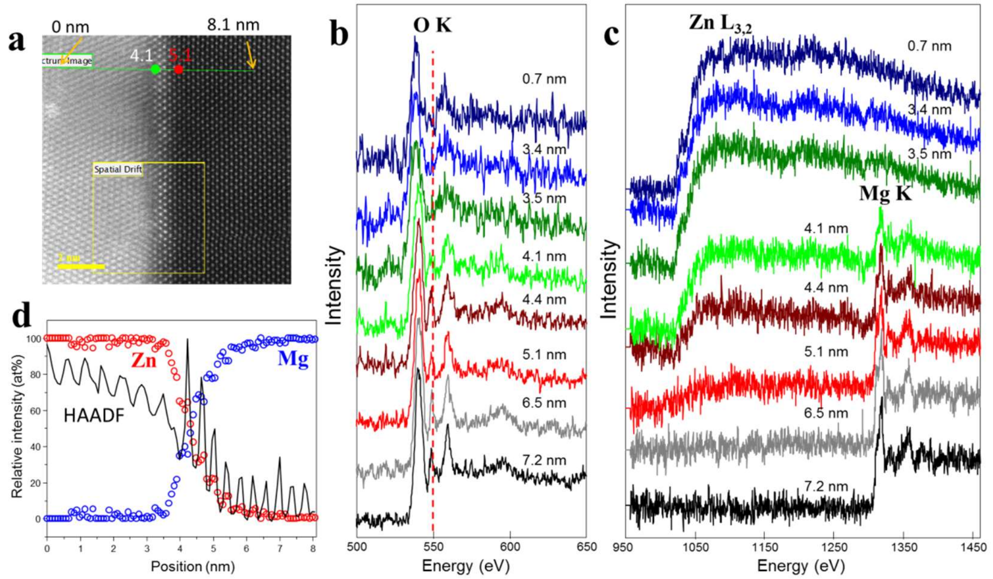

Supplementary Figure 9 (a) The scanning transmission electron microscopy (STEM)-_high-angle annular dark-field (HAADF) survey image. The green line marks the scan line for the electron energy loss spectroscopy (EELS) spectrum image. (b, c) Simultaneously acquired dual EELS for (b) O K edge and (c) Zn L_{32} and Mg K edge over the interface. (d) Relative composition of Zn (red circles) and Mg (blue) calculated from the spectrum image from the scan line shown in (a). The STEM-HAADF intensity signal (black line) is included.

Supplementary Note 11. EELS simulated by DFT calculations for O K edge.

In order to investigate the electronic structure of the interphase, we calculated the EELS spectra by using the TELNES package included in the WIEN2K code[2] based on DFT for O K-edge from MgO with cubic structure to ZnO with wurtzite with an increasing Zn concentration. The evolution of the simulated EELS is consistent with the experimental results.

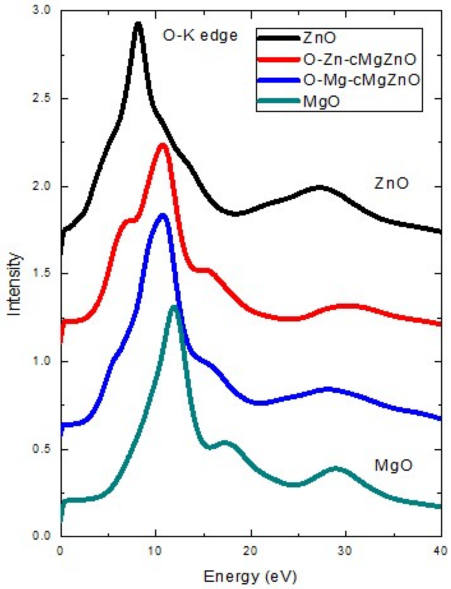

Supplementary Figure 10. Electron energy loss spectroscopy (EELS) simulated by density functional theory (DFT) calculations for O K edge. The blue and red spectra correspond to x=0.25 and x=0.75 for the cubic phase of $Mg_{1-x}Zn_xO$, respectively.

Supplementary Note 12. More details of first principles calculations

For first principles calculations, we used Vienna ab initio simulation package (VASP) based on the density functional theory (DFT) to calculate the surface energies, interface energies, adsorption energies, and diffusion barriers, etc. The exchange-correlation functional with Perdew-Burke-Ernzerh of generalized gradient approximation (GGA), projected augmented wave (PAW) potentials, and an energy cutoff of 500 eV were used in the calculations.

Supplementary Table 1 shows the lattice parameters and bulk modulus of ZnO and MgO obtained from our first principles calculation as well as data from literatures. Our calculated results are in good agreement with references. The free energy change per volume for Equations (Supplementary Equations 4 and 5) in Supplementary Note 18 is estimated using the energy difference between the ZnO bulk crystal and its constituent atoms (Zn and O) with the supercell approach, which yields a value of 0.353 eV/$Å^3$.

Supplementary Table 1 The lattice parameters and bulk modulus from this work and references for MgO and ZnO.

		MgO	ZnO
Lattice parameters (Å)	This work	a=4.251	a=3.280; c=5.298
	Reference	4.25[ref.9]	a=3.28[ref.10]; c=5.29[ref.10]<br>a=3.28[ref.11]; c=5.28[ref.11]
Bulk modulus (GPa)	This work	153	136
	Reference	169[ref.12], 156[exp-ref.13]	128[ref. 10], 129-146[ref.11]

Supplementary Note 13. Calculations of cleavage energy.

All surfaces were represented by periodically repeated slabs consisting of several atomic layers and separated by a vacuum region of more than 14 Å. All surface computations have been carried out based on DFT. The slabs are relaxed until the force on the ions is less than 0.004eV/Å. Good convergence has been achieved by considering suitable *k* points and number of atomic layers. The issue of internal electric field of ZnO c-plane surface has been addressed according to the strategies and discussions by Meyer[14] and Wander *et al*[15], and the results of slab calculations have been extrapolated containing up to 20 layers. Supplementary Table 2 shows the cleavage energies from this work with different number of atomic layers and those values from references for comparison. Our results suggest that the well converged cleavage energy can be achieved with a certain number of atomic layers, and the obtained values of cleavage energy are in good agreement with available data in literatures. Surface energies of MgO and ZnO can be considered to be half of the values of cleavage energies shown in Supplementary Table 2 (using the results with most layers calculated).

Supplementary Table 2 Cleavage energies from the different planes with different atomic layer number, comparing with data from references.

		(001) MgO	c-plane ZnO	m-plane ZnO	a-plane ZnO	FCC ZnO
Cleavage energy (eV/Å^2)	This work	4 layers: 0.112	12 layers: 0.215	6 layers: 0.110	6 layers: 0.116	6layers 0.065
		6 layers: 0.112	20 layers: 0.213	10 layers: 0.111	10 layers: 0.118	
	Reference	0.113[ref.16] 0.125[ref.17]	0.219[ref.10] 0.213[ref.18]	0.113[ref.10] 0.150[ref.18]	0.119[ref.10] 0.138[ref.18]	--

Supplementary Note 14. Calculations of interface energies.

The interface energy of ZnO/MgO was obtained through the calculations of surface energies of ZnO and MgO, as well as the adhesion energy of ZnO and MgO slabs, based on DFT. The relationship among interface energy, surface energy, and adhesion energy can be expressed[19] as $\gamma=\gamma_{s1}+\gamma_{s2}-\Delta\gamma$, where γ_{s1} is surface energy of the substrate (MgO), γ_{s2} is surface energy of the film (ZnO), and $\Delta\gamma$ the adhesion energy of the film on the substrate. Surface energies of MgO and ZnO are chosen to be half of the values of cleavage energies shown in Supplementary Table 2 (using the results with most layers calculated). Here, the adhesion energies and the resulted interface energies are listed in Supplementary Table 3. Additionally, for the m-plane interface, the case with a buffer phase consisting of two layers is also considered, for which the substrate surface energy uses a range between that of the (001)MgO and that of the (001)FCC ZnO, leading to a range of interface energy of 0.080-0.057 eV/Å^2 (1.280 -0.904 J/m^2).

Supplementary Table 3. Adhesion energies and interface energies from the different interfacial structures.

Interface structure	c-plane interface with O-termination	c-plane interface with Zn-termination	m-plane interface
Adhesion energy	0.044 (eV/Å^2)<br>0.704 (J/m^2)	0.020 (eV/Å^2)<br>0.320 (J/m^2)	0.027 (eV/Å^2)<br>0.432 (J/m^2)
Interface energy	0.119 (eV/Å^2)<br>1.904 (J/m^2)	0.143 (eV/Å^2)<br>2.288 (J/m^2)	0.084 (eV/Å^2)<br>1.344 (J/m^2)

Supplementary Note 15. Calculations of interface azimuthal registry

We have also performed theoretical calculations for a range of interface structures with different azimuthal registry to compare with the experimental determinations of interfacial relationship for both c-ZnO/MgO and m-ZnO/MgO. Cluster models (with outmost layers of atoms saturated with hydrogen) instead of superlattice models are used to allow the flexible rotation of the epilayers of ZnO, as small rotational angles would lead to huge sizes of unit cell, which demands a significant amount of computation time. For the calculations of both c-ZnO / MgO and m-ZnO / MgO, a 4-layer thick slab size of the MgO substrate is used with a 4×4×2 cell consisting of 256 atoms with a vacuum region of 25Å. The c-ZnO cluster is chosen to consist of 115 atoms (39H+ 50O +26Zn), while that of m-ZnO consists of 142 atoms (45H+ 62O +35Zn). Setting the experimentally observed registry structure as the origin point (orientation degree = 0º), a series of other possible registry structures can be obtained by the clockwise (anti-clockwise) rotation of the ZnO overlayers along the surface normal of the MgO substrate, defined for negative (positive) rotation degrees. As shown in Supplementary Figures 11a and 11b, over the rotational degree range from -15° to +15°, the 0° (origin) point (for which the energy is set as the zero reference) shows the lowest energy values. The typical side view models for 0° and 15° rotations of c-ZnO cluster are shown in Supplementary Figures 11c and 11d, respectively, while those for -14° and 0° rotations of m-ZnO are shown in Supplementary Figures 11e and 11f, respectively. For the +15º rotation for c-ZnO / MgO (Supplementary Figure 11d), the registry structure reaches the interface relationship of [100][010](001)MgO//[100][-1-20](001)ZnO; for the -14º rotation for m-ZnO / MgO (Supplementary Figure 11e), the interfacial relationship becomes [100][010](001)MgO//[010][001](100)ZnO. The calculation results indicate that the experimentally observed interface relationship corresponds to a stable interface structure for both c-ZnO / MgO and m-ZnO / MgO cases.

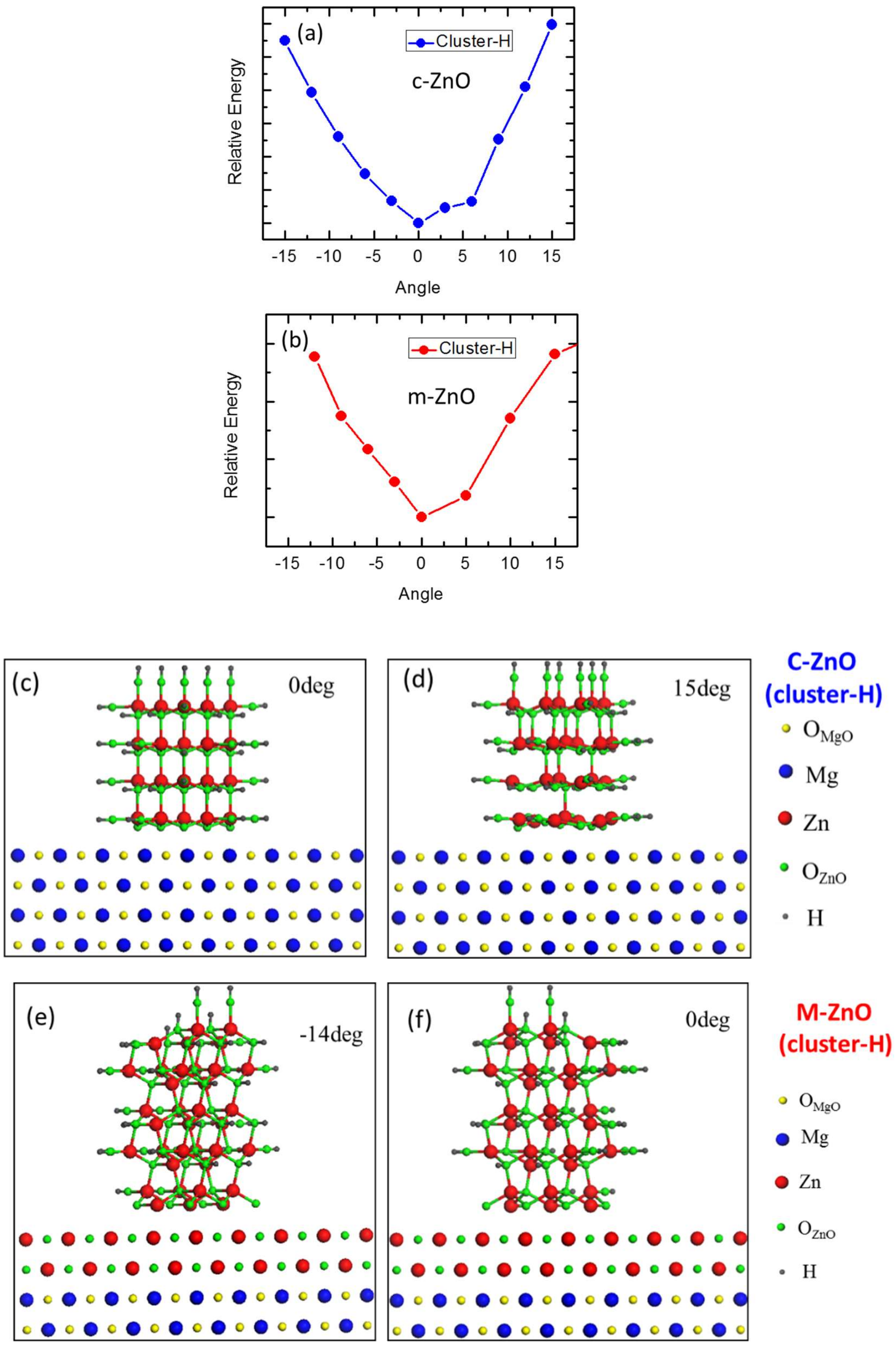

Supplementary Figure 11. Possibilities of azimuthal registry at the interface. (a) and (b) show relative energies of the different azimuthal registry models over a range of rotation degree from -15° to +15°. (The experimentally observed orientation is set as zero rotation degree as well as zero energy reference, for both c-ZnO and m-ZnO.) The atomic models of the typical azimuthal registry structures for c-ZnO and m-ZnO are shown in (c-d) and (e-f), respectively.

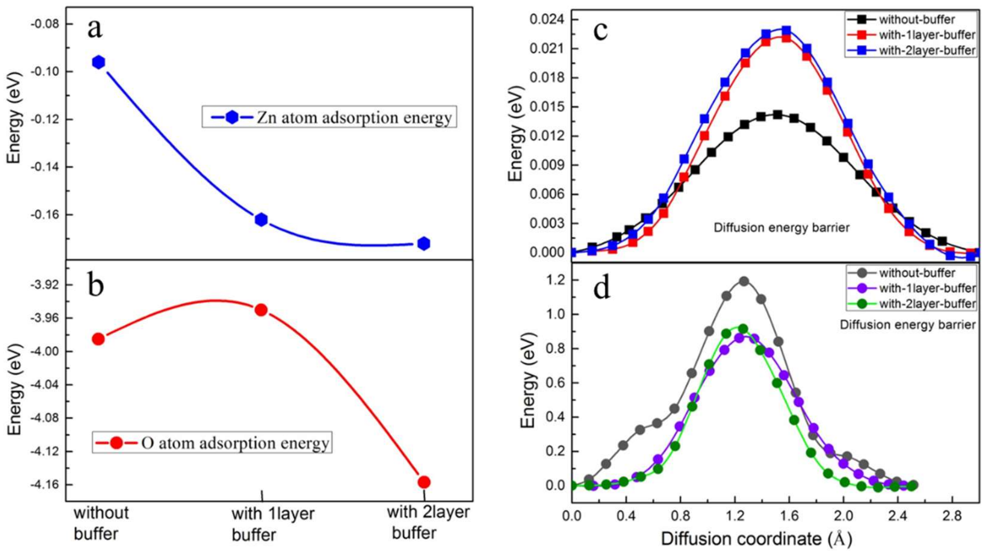

Supplementary Figure 12. (a) and (b) adsorption energies of the Zn and O atoms, respectively, on the MgO substrate without and with buffer layers; (c) and (d) the corresponding Zn and O atom diffusion barrier energy, respectively, on the MgO (001) surface without and with buffer layers.

Supplementary Note 16. Calculations of adsorption energy and diffusion barriers.

In order to investigate the growth mechanism, we calculated the adsorption energies and diffusion barriers for O and Zn atoms on the ideal MgO (001) surface and on the MgO (001) surface with the $Zn_xMg_{1-x}O$ buffer phases. We built the MgO (001) surface structures with more than 4 layers and with a vacuum distance of more than 14 Å. Supplementary Figure 13 displays the corresponding atomic models. The Monkhorst-Pack *k*-point mesh of 6×6×2 was used for these calculations. The convergence force on each ion was less than 0.05 eV/Å. The calculation of the adsorption energy follows the formula: $E_{adsorption}=E_{total}-E_{bare}-E_{atomic\text{-}adtom}$,[20] where E_{total} and E_{bare} are the energy of the system after and before adsorption, respectively and $E_{atomic\text{-}adtom}$ is the energy of an adtom. The diffusion energy barriers are calculated in conjunction with the climbing image (CI) nudged elastic band method (NEB).[21,22]

We calculated the Zn atom adsorption energy for three saddle-point configurations, namely, Zn atom sitting on the top of O, on the top of metal atom, or on the bridge position between next nearest atoms, respectively, as shown in the Supplementary Figures 13(a-i). While for the O atoms (Supplementary Figures 13 (j-q)), the lowest-energy configuration appears at the tilt position closed to the O atom top after relaxation, as illustrated in the Supplementary Figures 13 (k, n, q). All adsorption energies are shown under their corresponding images in Supplementary Figure 13. The adsorption energies of the most stable saddle-point configurations for Zn and O atoms are plotted in Supplementary Figures 12a and 12b, respectively, comparing the cases without and with the buffer layer. Supplementary Figures 12c and 12d show the diffusion energy barriers for Zn and O atoms, respectively, also for the cases without and with the buffer layer.

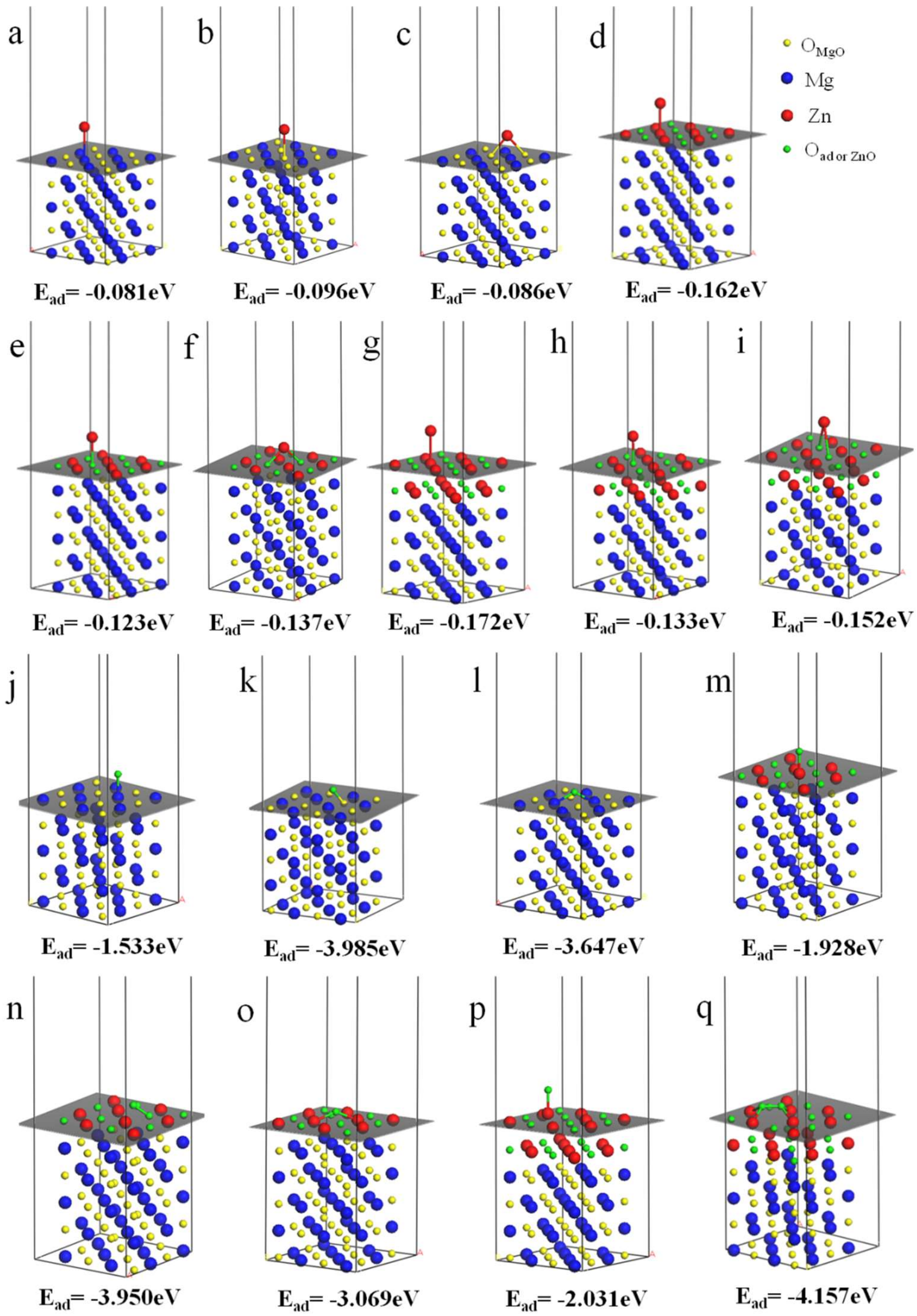

Supplementary Figure 13. Three dimensional (3D) atomic models of different saddle-point configurations for Zn and O adatoms. Zn adatoms (a-i) and O adatoms (j-l) on the MgO (001) surface, respectively; (d-f) and (g-i) Zn adatoms on the 1-layer and 2-layer buffer layers with face-centered structure, respectively; (m-o) and (p, q) O adatoms on the 1-layer and 2-layers buffer layers, respectively.

Supplementary Note 17. In-situ annealing experiments.

Supplementary Figure 14a shows the *in-situ* RHEED pattern from the ZnO film after the deposition for 10 min at the growth temperature of~120 °C with an O_2 pressure of 2×10^{-5} mbar and a plasma of 200 *w*. The blue and red dashed rectangles represent the diffraction dots of the reciprocal lattice space of the (110)-plane and (100)-plane, respectively. And the dim RHEED pattern is due to the small nucleation at this stage. Supplementary Figure 14 (b,c) show the *in-situ* RHEED patterns after annealing at the about ~400°C without O_2 and with O_2, respectively. It appears that the surface of ZnO film becomes flatter after annealing without O_2, and become rough again after annealing with O_2. However, there appears no change of the orientation of the ZnO film during the annealing processes at ~400°C.

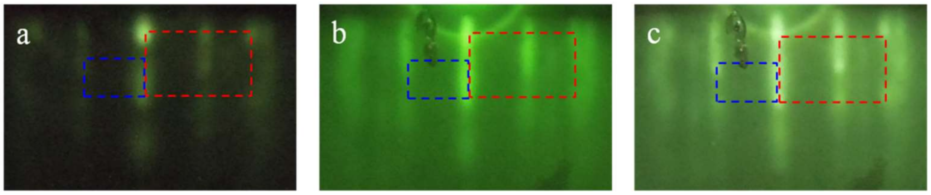

Supplementary Figure 14. In-situ reflection high energy electron diffraction (RHEED) patterns from the ZnO films. (a) after growing for 10 min; (b) after annealing for 30 min without O_2 at ~400 °C; (c) annealing for 30 min with O_2 at ~400 °C.

Supplementary Note 18. Contact angle of nucleation and nucleation barrier of c-ZnO and m-ZnO films on the MgO substrate

Based on the classical theory of nucleation[3-5], the overall excess free energy, ΔG, can be expressed as,

$$\Delta G=\Delta G_S+\Delta G_V \qquad \text{(Supplementary Equation 2)}$$

where ΔG_S is the excess free energy between the surface of the particle and the bulk of the particle, and ΔG_V is the free energy change of the transformation. This treatment for the nucleating process can be extended to the formations of cluster or island on supporting substrates at the initial stage of film growth[6]. For nuclei in shape of clusters or islands that can be described in terms of macroscopic surface energy, the form of ΔG for island is given by

$$\Delta G= S_{\text{interface}}\cdot(\gamma_{\text{interface}} - \gamma_{\text{substrate}}) + S_{\text{island}}\cdot\gamma_{\text{island}} + V_{\text{island}}\cdot\Delta G_v \qquad \text{(Supplementary Equation 3)}$$

where $S_{\text{interface}}$ is the interface area of contact between the nucleation island and the substrate, S_{island} and V_{island} are the surface area and volume of nucleation island, respectively. $\gamma_{\text{interface}}$ represents the interface energy per unit area, and $\gamma_{\text{substrate}}$ and γ_{island} are the surface energy per unit area for substrate and island, respectively. ΔG_v is the free energy change of the island per unit volume.

For the ZnO islands grown on the MgO (001) substrate, it is possible to form island with growth orientation of c-plane or m-plane, depending on growth conditions, as aforementioned. Therefore, we distinguish these two cases by labeling "c" and "m" as the subscript in the terms of free energy, as shown below,

$$\Delta G_c= S_{\text{c,interface}}\cdot(\gamma_{\text{c,interface}} - \gamma_{\text{substrate}}) + S_{\text{c,island}}\cdot\gamma_{\text{c,island}} + V_{\text{c,island}}\cdot\Delta G_v \qquad \text{(Supplementary Equation 4)}$$

for c-ZnO, and

$$\Delta G_m= S_{\text{m,interface}}\cdot(\gamma_{\text{m,interface}} - \gamma_{\text{substrate}}) + S_{\text{m,island}}\cdot\gamma_{\text{m,island}} + V_{\text{m,island}}\cdot\Delta G_v \qquad \text{(Supplementary Equation 5)}$$

for m-ZnO.

The interface energy per unit area ($\gamma_{\text{c,interface}}$ and $\gamma_{\text{m,interface}}$), surface energy per unit area ($\gamma_{\text{substrate}}$), and free energy change per unit volume (ΔG_v) can be obtained through first principles calculations. The estimation of S and V, as well as $\gamma_{\text{c,island}}$ and $\gamma_{\text{m,island}}$ depends on the morphology of the islands. The typical morphologies of c-ZnO and m-ZnO islands are displayed in Supplementary Figure 15, and the typical contact angle for c-ZnO is about 11°, and that for m-ZnO is about 3°.

In the following we will show, from complex geometry (frustum with facets or circular cone frustum) to simplified geometry (spherical caps), the comparison of free energy and nucleation barrier of c- and m-ZnO islands as a function of size.

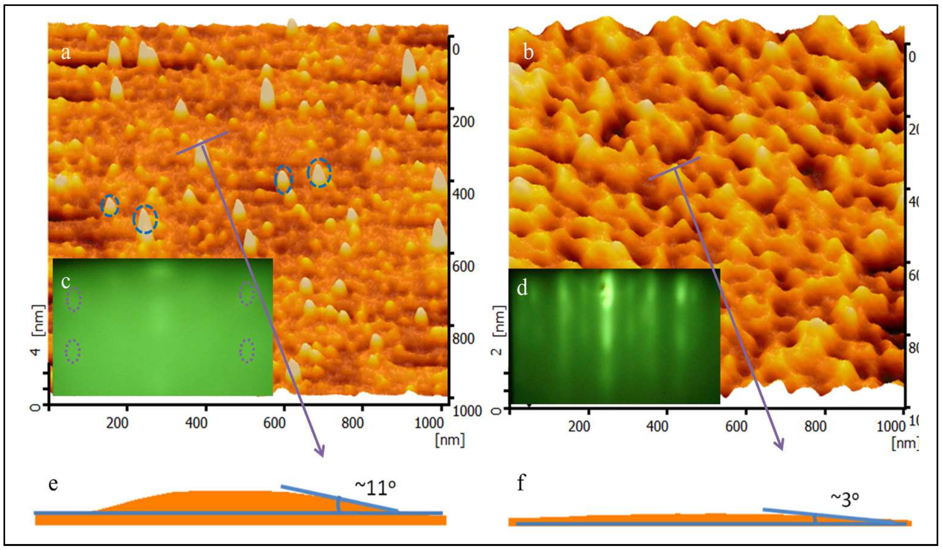

Supplementary Figure 15. Morphology of c-ZnO and m-ZnO islands. (a) AFM image of c-ZnO. (b) AFM image of m-ZnO. (c) RHEED pattern of c-ZnO, and (d) RHEED pattern of m-ZnO. Typical profile lines of island in AFM image for (e) c-ZnO, and (f) m-ZnO with the same scale of size in unit of nm. The typical contact angle for c-ZnO is about 11°, and that for m-ZnO is about 3°.

Supplementary Note 19. Morphology of islands in shape of frustum with facets

Assuming that the morphology of islands in shape of frustum with facets can be approximated by a right frustum whose bases are regular *n*-sided polygons, as shown in Supplementary Figure 16, one can calculate the surface area and volume of the shape as below,

$S_{c,interface} = \frac{3\sqrt{3}}{2} a_{c2}^2,$ (Supplementary Equation 6)

$S_{c,island}$ (top surface)$= \frac{3\sqrt{3}}{2} a_{c1}^2,$ (Supplementary Equation 7)

$S_{c,island}$ (side surface)$= \frac{3}{2}\sqrt{(a_{c2}^2 - a_{c1}^2)^2 \sec^2\frac{\pi}{6} + 4h_c^2(a_{c1}+a_{c2})^2},$ (Supplementary Equation 8)

$V_{c,island} = \frac{h_c}{2}(a_{c1}^2 + a_{c1}a_{c2} + a_{c2}^2)\cot\frac{\pi}{6},$ for c-ZnO; (Supplementary Equation 9)

$S_{m,interface} = a_{m2}^2,$ (Supplementary Equation 10)

$S_{m,island}$ (top surface) $= a_{m1}^2,$ (Supplementary Equation 11)

$S_{m,island}$ (side surface) $= \sqrt{(a_{m2}^2 - a_{m1}^2)^2 \sec^2\frac{\pi}{4} + 4h_m^2(a_{m1}+a_{m2})^2},$ (Supplementary Equation 12)

and

$V_{m,island} = \frac{h_m}{3}(a_{m1}^2 + a_{m1}a_{m2} + a_{m2}^2)\cot\frac{\pi}{4},$ for m-ZnO. (Supplementary Equation 13)

By defining $a_{c1}=\alpha a_{c2}$, $a_{m1}=\alpha a_{m2}$, using the relationship $a_{c2} = r$, $a_{m2} = \sqrt{2}r$, $h_c = (a_{c2} - a_{c1})\tan\theta$, $h_m = (a_{m2} - a_{m1})\tan\theta$, one can rewrite the above equations of surface areas and volume in terms of r, θ, and α. If the morphology of islands shown in AFM images can be regarded as the typical geometry for our case, *i.e.*, using the parameters of $\alpha = 0.5$ for both orientations (a range of $\alpha = 0$-0.8 does not alter the conclusions), and contact angles of $\theta = 11°$ for c-ZnO and $\theta = 3°$ for m-ZnO, the surface areas and volume can be expressed as a function of characteristic radius, r.

For the energy terms, the interface energy, surface energy of MgO and top surface energy of c-ZnO and m-ZnO, as well as free energy change of the island (see Supplementary Note 12-16) can be used for estimating the free energy. The only unknown surface energy is the side (facet) surface energy. According to Wulff theorem [7-8], γ_i/R_i = constant, we have

$\gamma_{c,island}$(top surface)$/R_c = \gamma_{c,island}$(side surface)$/R_{c,side}$, (Supplementary Equation 14)

and

$\gamma_{m,island}$(top surface)$/R_m = \gamma_{m,island}$(side surface)$/R_{m,side}$. (Supplementary Equation 15)

Considering that the contact angles θ_c and θ_m are small, one may use the approximations $R_{c,side}/R_c \sim \sec\theta_c$, and $R_{m,side}/R_m \sim \sec\theta_m$, then

$\gamma_{c,island}$(side surface) $= \gamma_{c,island}$(top surface)$\cdot \sec\theta_c = \gamma_{c,island} \cdot \sec\theta_c$. (Supplementary Equation 16)

Similarly,

$\gamma_{m,island}$(side surface) $= \gamma_{m,island}$(top surface)$\cdot \sec\theta_m = \gamma_{m,island} \cdot \sec\theta_m$. (Supplementary Equation 17)

Combing the above equations, one can express the free energy $\Delta G_c(r)$ and $\Delta G_m(r)$ in terms of the characteristic radius, r, namely, the size of the nucleation island. Therefore, the nucleation barrier ΔG_c^* and ΔG_m^* can be derived by taking $d(\Delta G_c(r))/dr = 0$ and $d(\Delta G_m(r))/dr = 0$. The numerical simulation of free energy $\Delta G_c(r)$ and $\Delta G_m(r)$ and nucleation barriers ΔG_c^* and ΔG_m^* have been shown in Supplementary Figure 16c (the interface energy and surface energy obtained by first principles calculations shown in Supplementary Table 2 and 3 are used for numerical simulations). It can be seen that, at a large radius, c-ZnO has a lower free energy and nucleation barrier than that of m-ZnO, consistent with our experimental observations, as described in the main text.

Supplmentary Note 20. Morphology of islands in shape of circular cone frustum

Assuming that the morphology of islands is in shape of circular cone frustum, as shown in Supplementary Figure 17, then the surface area and volume of the shape can be obtained (in general form) as

$S_{\text{interface}} = \pi r^2$, (Supplementary Equation 18)

S_{island} (top surface) $= \pi\alpha^2 r^2$, (Supplementary Equation 19)

S_{island} (side surface) $= \pi(\alpha r+r)l = \pi r^2(1+\alpha)(1-\alpha)\sec\theta$, (Supplementary Equation 20)

$V_{,\text{island}} = \frac{1}{3}\pi h(\alpha^2 r^2+r^2+\alpha r^2) = \frac{1}{3}\pi r^3(1-\alpha)(1+\alpha+\alpha^2)\tan\theta$ (Supplementary Equation 21)

Again, using Wulff theorem[7-8], γ_i/R_i = constant, we have,

$\gamma_{\text{island}}(\text{side surface}) = \gamma_{\text{island}}(\text{top surface})\frac{R_{\text{side}}}{R} \sim \gamma_{\text{island}}(\text{top surface})\sec\theta_c$. (Supplementary Equation 22)

Combining the above equations with Eqn. (Supplementary Equations 4 and 5), one can calculate the free energy and nucleation barriers, as shown in Supplementary Figure 17. The trend of free energy of islands in shape of circular cone frustum is similar to that in shape of frustum with facets.

Supplementary Note 21. Morphology of islands in shape of spherical cap

Assuming that the morphology of islands is in shape of spherical cap, as shown in Supplementary Figure 18, then the surface area and volume of the shape can be obtained (in general form) as

$S_{\text{interface}} = \pi r^2$, (Supplementary Equation 23)

$S_{\text{island}} = 2\pi r^2(1-\cos\theta)(\csc\theta)^2$, (Supplementary Equation 24)

$V_{,\text{island}} = \frac{1}{6}\pi r^3(1-\cos\theta)\csc\theta\,(3+(1-\cos\theta)^2(\csc\theta)^2)$. (Supplementary Equation 25)

Combining equations (Supplementary Equations 4 and 5, 23-25), one can calculate the free energy and nucleation barriers, as shown in Supplementary Figure 18. The trend of free energy of islands in shape of spherical cap is similar to that in shape of circular cone frustum.

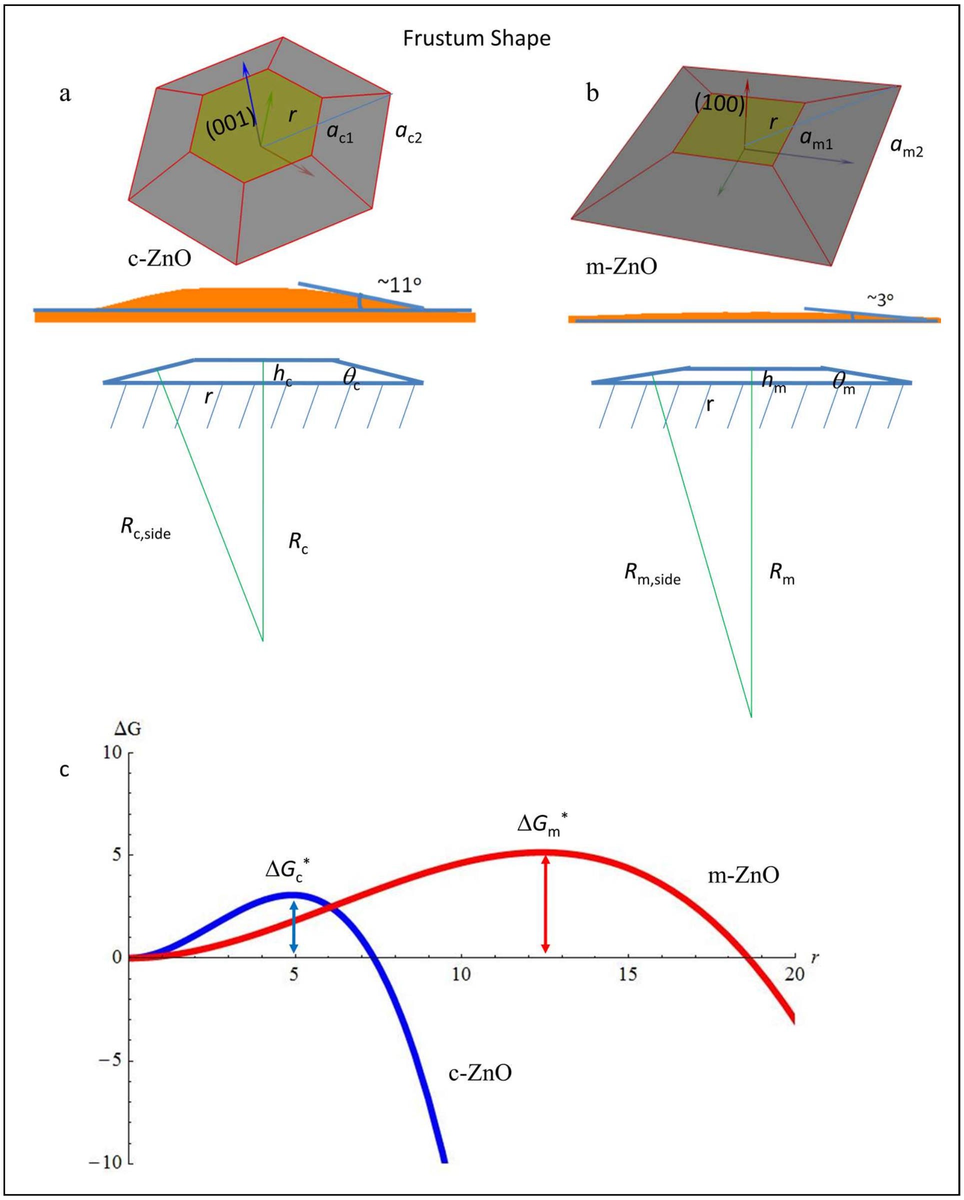

Supplementary Figure 16. Model of morphology of c-ZnO and m-ZnO islands in the frustrum shape. (a) c-ZnO, and (b) m-ZnO. The labels a_{c1} (a_{m1}) and a_{c2} (a_{m2}), h_c (h_m), R_c (R_m) and $R_{c,side}$ ($R_{m,side}$), are the edges of the two bases of c-ZnO (m-ZnO) islands, the height of island, the distance from the (possibly hypothetical) center of the crystal to a given set of facets. The characteristic radius of the island is labeled by r. (c) Free energy of c-ZnO and m-ZnO nucleation as a function of size. The nucleation barriers ΔG_c^* and ΔG_m^* have been indicated in the figure. With a contact angle of 11° for c-ZnO, the possible index of facets could be {109}, and with a contact angle of 3° for m-ZnO, the possible index of facets could be (10,0,±1) and (16, ±1,0).

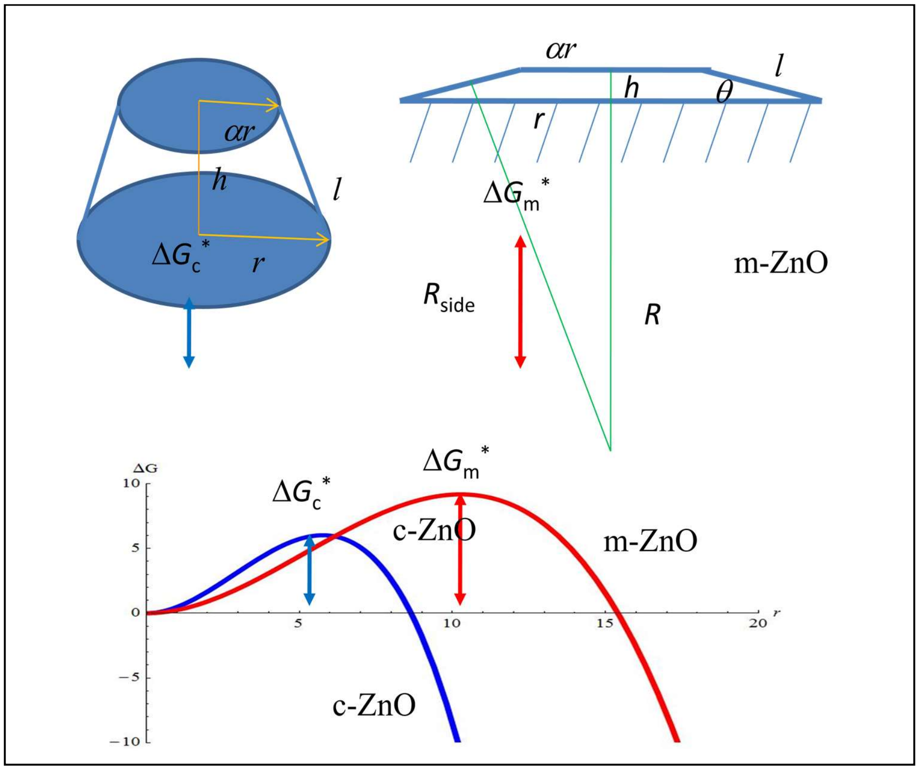

Supplementary Figure 17. Model of morphology of c-ZnO and m-ZnO islands in the circular cone frustrum shape. The characteristic radius of the island is labeled by r. The plots in the bottom show free energy of c-ZnO and m-ZnO nucleation as a function of size.

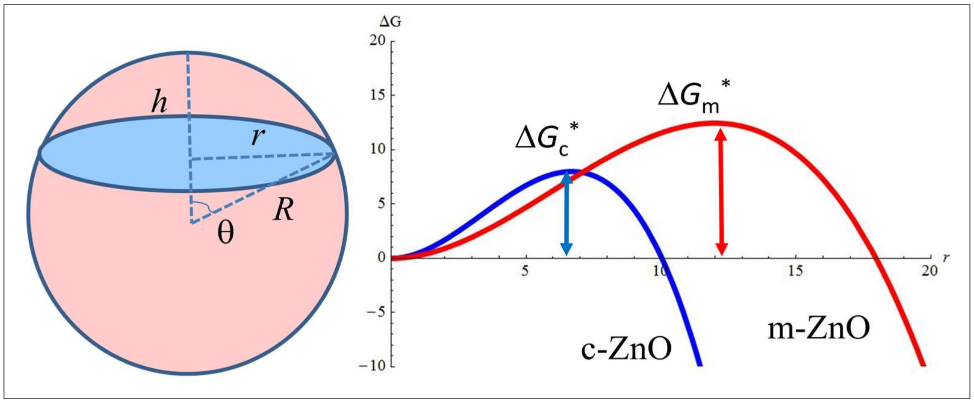

Supplementary Figure 18. Model of morphology of c-ZnO and m-ZnO islands in the spherical cap shape. The plots shown on the right-hand side are free energy of c-ZnO and m-ZnO nucleation as a function of size.

Supplementary References:

1. Grundmann, M.; Böntgen, T.; Lorenz, M. Occurrence of Rotation Domains in Heteroepitaxy. *Phys. Rev. Lett.* ***105***, 146102 (2010).
2. Blaha, P. *et al.*, *WIEN2K, An Augmented Plane Wave + Local Orbitals Program for Calculating Crystal Properties* (Technische Universitat Wein, Wein, 2001).
3. Mullin, J. W. *Crystallization* (Butterworth-Heinemann, Oxford, 4th edition, 2001).
4. Kalikmanov, V. I. *Nucleation Theory* (Springer, Dordrecht, 2013).
5. Oxtoby, D. W. Nucleation of first-order phase transitions. *Acc. Chem. Res.* 31, 91-97 (1998).
6. Venables, J. A., Spiller, G. D. T., & Hanbucken M. Nucleation and growth of thin films. *Rep. Prog. Phys.* **47,** 399-459 (1984).
7. Wulff G., Zur Frage der Geschwindigkeit des Wachstums und der Auflösung der Kristallflächen (Regarding the question of the growth rate and dissolution of crystal surfaces)*, Zeitschrift für Kristallographie (Crystalline Materials)* **34**, 449 (1901).
8. Dinghas A., Über einen geometrischen Satz von Wulff für die Gleichgewichtsform von Kristallen (About a Wulff geometric model for the equilibrium shape of crystals)*, Zeitschrift für Kristallographie (Crystalline Materials)* **105**, 304 (1944).
9. Skorodumova, N. V., Hermansson, K. & Johansson, B. Structural and electronic properties of the (100) surface and bulk of alkaline-earth metal. *Phys. Rev. B* **72,** 125414 (2005).
10. Meyer, B. & Marx, D. Density-functional study of the structure and stability of ZnO surfaces. *Phys. Rev. B* **67,** 035403 (2003).
11. Sponza, L., Goniakowski, J. & Noguera, C. Structural, electronic, and spectral properties of six ZnO bulk polymorphs. *Phys. Rev. B* **91,** 075126 (2015).
12. Jaffe, J. E., Snyder, J. A., Lin, Z. & Hess, A. C. LDA and GGA calculations for high-pressure phase transitions in ZnO and MgO. *Phys. Rev. B* **62,** 1660(2000).
13. Mao, H. K. & Bell,P. M. Equations of state of MgO and ε Fe under static pressure conditions. *J. Geophys. Res.* **84**, 4533 (1979).
14. Meyer, B.First-principles study of the polar O-terminated ZnO surface in thermodynamic equilibrium with oxygen and hydrogen, *Phys. Rev. B* **69,** 045416 (2004).
15. Wander A. and Harrison N. M., The stability of polar oxide surfaces: The interaction of H_2O with ZnO(0001) and ZnO(000-1). *J. Chem. Phys.***115,** 2312-2316 (2001).
16. Skorodumova, N. V., Hermansson, K., & Johansson, B. Structural and electronic properties of the (100) surface and bulk of alkaline-earth metal. *Phys. Rev. B* **72,** 125414 (2005).
17. Broqvist, P., Grönbeck, H., & Panas, I. Surface properties of alkaline earth metal oxides. *Surf. Sci.* **554,** 262–271 (2004).
18. Diebolda, U., Koplitzb, L. V., Duluba, O. Atomic-scale properties of low-index ZnO surfaces. *Appl. Surf. Sci.* **237,** 336–342 (2004).
19. Henry, C. R. Morphology of supported nanoparticles. *Prog. Surf. Sci.* **80**, 92-116 (2005).
20. Xu H., Zhang R. Q., and Tong S. Y, Interaction of O_2, H_2O, N_2, and O_3 with stoichiometric and reduced ZnO

(10-10) surface, *Phys. Rev. B* **82**, 155326 (2010).

21. Henkelman G., Uberuaga B. P., and Jónsson H., A climbing image nudged elastic band method for finding saddle points and minimum energy paths. *J. Chem. Phys.* **113**, 9901 (2000).
22. Erhart P. and Albe K., First-principles study of migration mechanisms and diffusion of oxygen in zinc oxide. *Phys. Rev. B* **73**, 115207 (2006).
